# Challenges for Pulsed Laser Deposition of FeSe Thin Films

**DOI:** 10.3390/mi12101224

**Published:** 2021-10-07

**Authors:** Yukiko Obata, Igor A. Karateev, Ivan Pavlov, Alexander L. Vasiliev, Silvia Haindl

**Affiliations:** 1 Tokyo Tech World Research Hub Initiative (WRHI), Institute of Innovative Research, Tokyo Institute of Technology, 4259 Nagatsuta-cho, Midori-ku, Yokohama, Kanagawa 226-8503, Japan; 2 National Research Centre “Kurchatov Institute”, pl. Akademika Kurchatova 1, 123182 Moscow, Russia; iakarateev@gmail.com; 3 Shubnikov Institute of Crystallography of FSRC “Crystallography and Photonics” Russian Academy of Sciences, Leninsky pr. 59, 119333 Moscow, Russia; win8765495@gmail.com; 4 Moscow Institute of Physics and Technology, National Research University, Dolgoprudny, 141701 Moscow, Russia

**Keywords:** PLD, Fe-based superconductor, FeSe, thin film, TEM

## Abstract

Anti-PbO-type FeSe shows an advantageous dependence of its superconducting properties with mechanical strain, which could be utilized as future sensor functionality. Although superconducting FeSe thin films can be grown by various methods, ultrathin films needed in potential sensor applications were only achieved on a few occasions. In pulsed laser deposition, the main challenges can be attributed to such factors as controlling film stoichiometry (i.e., volatile elements during the growth), nucleation, and bonding to the substrate (i.e., film/substrate interface control) and preventing the deterioration of superconducting properties (i.e., by surface oxidization). In the present study, we address various technical issues in thin film growth of FeSe by pulsed laser deposition, which pose constraints in engineering and reduce the application potential for FeSe thin films in sensor devices. The results indicate the need for sophisticated engineering protocols that include interface control and surface protection from chemical deterioration. This work provides important actual limitations for pulsed laser deposition (PLD) of FeSe thin films with the thicknesses below 30 nm.

## 1. Introduction

Iron-based superconductor FeSe thin films haven been grown by different methods [1,2]: pulsed laser deposition (PLD) [3,4,5,6], molecular-beam epitaxy (MBE) [7], radio-frequency (RF) sputtering [8,9], selenization [10], and mechanical exfoliation [11]. So far, high-superconducting transition temperature (*T*_c_) monolayers as well as superconducting ultrathin FeSe films could be obtained in an MBE approach, for example, one-unit-cell FeSe/SrTiO_3_(STO) with *T*_c_ = 64–75 K [12,13,14,15,16], or 1–5 nm thin FeSe films on bilayer graphene with *T*_c_ = 3–8 K [7]. There, *T*_c_ values are usually extracted from in situ measurements such as scanning tunneling microscopy and angle-resolved photoemission spectroscopy. Mechanical exfoliation has recently succeeded in producing superconducting FeSe sheets with the thickness between 15–60 nm and *T*_c_ = 6–7 K [11] or even a thickness of 10 nm with *T*_c_ = 5.2 K [17], measured by resistive measurements under ambient conditions. However, at present it is not clear what is the minimum thickness of exfoliated thin FeSe films, which can technically be established. A superconducting transition was also found in RF-sputtered FeSe films with thicknesses above 20 nm [8].

By means of PLD ~20 nm thin superconducting films were produced either on CaF_2_ substrates [18,19] or by post-annealing [20]. Recently, ~33 nm thin FeSe films have been grown by PLD on Fe/MgO with *T*_c_ = 4 K [21]. The strong relationship between structural and electronic properties in thin films of FeSe could be exploited in future electronic devices and sensor applications [4,22,23,24,25]. Unfortunately, a successful growth of ultrathin (<10 nm), superconducting FeSe thin films using PLD has not yet been reported. Most available data for FeSe thin films on MgO are listed in Table 1, which demonstrates a wide spread in *T*_c_ from not superconducting to a maximum 8 K (bulk value), and a strong decrease in *T*_c_ with decreasing film thickness. In addition, there are hardly consistent reports about composition of targets and films. Its challenge can be attributed to several factors: (1) Control of stoichiometry, i.e., the control of volatile elements during target ablation, plume expansion, and film nucleation. The volatility of Se may prevent stoichiometric transfer of the material from target to substrate. (2) The control of the chemical bonding between film and substrate, as well as film growth modes and film strain. (3) Preventing the deterioration of the film surface (for example, surface oxidization). 

In the present study, we address these technical issues in the growth of FeSe thin films in examples of FeSe films on MgO and mica substrates. In particular, we have investigated the effect of substrate pretreatment and post-annealing under Se vapor. We also studied Fe buffer and overlayers with configurations of FeSe/Fe/MgO and Fe/FeSe/MgO and discuss growth, structural parameters, and electronic properties of FeSe. We found that a substrate pretreatment in Se vapor or the incorporation of Fe buffer or overlayers have an effect on the structural (and electronic) parameters. Furthermore, similar structural parameters were obtained for FeSe/Fe/MgO and Fe/FeSe/MgO films, leading both to an incipient superconducting transition in FeSe. In contrast to Ref. [20], however, we do not find superconductivity induced after post-annealing, neither in UHV nor under Se vapor.

## 2. Methods

### 2.1. Thin Film Deposition

FeSe thin films were grown on single crystalline MgO(001) (10 × 10 × 0.5 mm^2^, Furuuchi Chemical Co., Tokyo, Japan) or mica(001) (i.e., KMg_3_AlSi_3_O_10_F_2_, 10 × 10 × 0.5 mm^2^, Crystal Base Co., Osaka, Japan) by pulsed laser deposition (PLD) using an EV-100/PLD-S growth chamber (Eiko, Tokyo, Japan) under a base pressure smaller than 1 × 10^−6^ Pa. MgO substrates were heated in air at 800 °C for 2 h before they were inserted into the UHV chamber. Mica substrates were inserted without processing. A KrF excimer laser (COMPex Pro 110F, Coherent GmbH, Göttingen, Germany, λ = 248 nm) was used for ablating the FeSe, Fe, and Au targets with the laser energy density of 2 Jcm^−2^ for FeSe and 3 Jcm^−2^ for Fe and Au. The distance between target and substrate during deposition was approximately 5 cm. The substrate heating was achieved by an infrared semiconductor laser (LU0915C300-6, Lumics GmbH, Berlin, Germany, λ = 915 nm). The deposition of FeSe was always performed at 350 °C at a repetition rate of 10 Hz (a typical growth rate of ~0.25 Ås^−1^). In two cases, the repetition rate of 5 Hz was chosen to achieve the low growth rate of ~ 0.05 Ås^−1^. The film thickness was typically 30 nm. To perform PLD, FeSe targets were fabricated via solid-state reaction, as described in Ref. [21]. Rietvelt refinement of the powder XRD of the targets reveals the lattice parameters of *a*_FeSe_ = 3.771(1) Å and *c*_FeSe_ = 5.521(1) Å. The deposition of Fe and Au was performed at room temperature and a repetition rate of 10 Hz from elemental commercial targets, respectively.

**Preparation of FeSe/Fe/MgO**: Fe buffer layers were grown with a thickness of 5–15 nm. The Fe-layer was annealed at *T_S_*_,Fe_ = 600 or 650 °C for 5 min and cooled to the deposition temperature for FeSe at 350 °C. FeSe films were kept at the temperature for 30 min prior to deposition and fabricated at a repetition rate of 10 Hz.

**Preparation of Fe/FeSe/MgO**: After the deposition of FeSe at 350 °C, the films were cooled down to room temperature, and the Fe overlayers were deposited. In case of Au-capping, a 20 nm thin Au cap layer was prepared in situ.

**Pre-annealing of substrates in the chamber**: MgO substrates were annealed at 800 °C for 30 min in UHV or in Se vapor and afterwards brought to the deposition temperature of 350 °C. In case of Se vapor annealing, Se (Kojundo, 99.99%) was evaporated at 150 °C from a Knudsen cell (K-cell), and its flux was controlled by the cell temperature with a beam flux monitor positioned just below the substrate. The Se vapor pressure was kept at ~ 1 × 10^−5^ Pa during the process. After 30 min, the substrates were cooled down to room temperature after their heat treatment either in UHV or in Se vapor. Then, the Fe buffers were deposited with a thickness of 6–8 nm. The Fe-covered MgO was heated to annealing temperatures, *T_S_*_,Fe_, of either 600 °C or 650 °C, held at this temperature for 5 min, and subsequently cooled to the deposition temperature for FeSe at 350 °C. FeSe films were kept at the temperature for 30 min prior to deposition and fabricated at a repetition rate of 10 Hz.

**Post-annealing of the films in the chamber**: Films were annealed in situ at 400 °C for 30 min in UHV or in Se vapor. An annealing temperature of 400 °C was chosen because post-annealing at 450 °C resulted in the appearance of FeSe(101) reflection in XRD patterns. In case of post-annealing in Se vapor, the Se K-cell was switched on and heated to 150 °C before film deposition. However, the shutter remained closed during deposition. A different, ex situ vapor transport annealing method was reported in [26].

### 2.2. Characterization

In order to investigate phase and crystal orientation of the films, we performed standard XRD 2θ/ω in Bragg Brentano geometry and high-resolution scans in parallel beam geometry, using a SmartLab diffractometer (Rigaku) with CuKα radiation. The typical step size Δ2θ of the scans was 0.02°. The c-axis lattice parameters were obtained from FeSe(00l) reflections with l = 1, 2, 3 and 4 by means of a linear extrapolation versus the Nelson-Riley function cos(θ)cot(θ) + cos^2^(θ)/λ [27]. The layer thicknesses of FeSe and Fe were determined by X-ray reflectivity (XRR) measurements using a SmartLab diffractometer (Rigaku) with CuKα1 monochromated by Ge(220).

Film/substrate interfaces and film cross sections were analyzed by transmission electron microscopy (TEM), scanning transmission electron microscope (STEM), and energy dispersive X-ray spectroscopy (EDXS). The specimens for the TEM, STEM, and EDXS studies were prepared by using a standard lift-out FIB technique in a Helios Nanolab focus ion beam (FIB) scanning electron microscope (SEM) (Thermo Fisher Scientific, Waltham, MA, USA). A Pt protective layer with the thickness of 1–2 µm was deposited on the specimen surface by electron beam following Ga+ ion beam deposition. Microstructural analyses were performed in a Titan 80–300 TEM/STEM (Thermo Fisher Scientific, Waltham, MA, USA) equipped with a spherical aberration corrector (probe corrector) with an accelerating voltage of 300 kV. Such a configuration allows one to obtain images in STEM mode with a resolution of 0.08 nm. The device is equipped with an EDX Si(Li) spectrometer (EDAX, Mahwah, NJ, USA), a high-angle annular dark-field (HAADF) electron detector (Fischione, Export, PA, USA) and a Gatan image filter (GIF) (Gatan, Pleasanton, CA, USA). In addition, some of the STEM images and EDXS data were obtained in an Osiris (Thermo Fisher Scientific, Waltham, MA, USA) at an accelerating voltage of 200 kV. This instrument is also equipped with an HAADF detector (Fischione, Export, PA, USA) and a silicon drift detector (SDD), i.e., a Super-X EDX detector (Bruker, USA). Image processing was performed using a Digital Micrograph (Gatan, Pleasanton, CA, USA) and TIA (FEI, Hillsboro, OR, USA) software.

AES depth profiles were acquired on thin films using an ULVAC-Phi 710 Auger electron spectrometer with an integrated scanning electron microscope and an Ar sputtering gun. The analysis was conducted using a focused electron beam with a primary energy of 10 keV and an electron current of 10 nA. The etching rate was ~2 nm⸱min^−1^ with an Ar^+^ ion primary energy of 1 keV on a square area of 1 × 1 mm^2^. In the preparation of the FeSe/MgO film for Auger electron spectroscopy (AES) depth profiling, Au capping was performed by sputtering. Here, Au was deposited at room temperature by direct current sputtering using an SPF-332HS sputtering system (Canon Anelva, Kanagawa, Japan).

The temperature dependence of the longitudinal resistivity of the films was measured with a physical property measurement system (Quantum Design Inc., San Diego, CA, USA) in the range of 2–300 K by the four-probe method. Silver paste was employed for electrical contacts. Only for one film, a 90% criterion could be employed for the determination of *T*_c_. These measurements were performed mostly within 10 days after film fabrication. The films were stored in an evacuated desiccator prior to the measurements.

## 3. Results and Discussion

**Figure 1:**
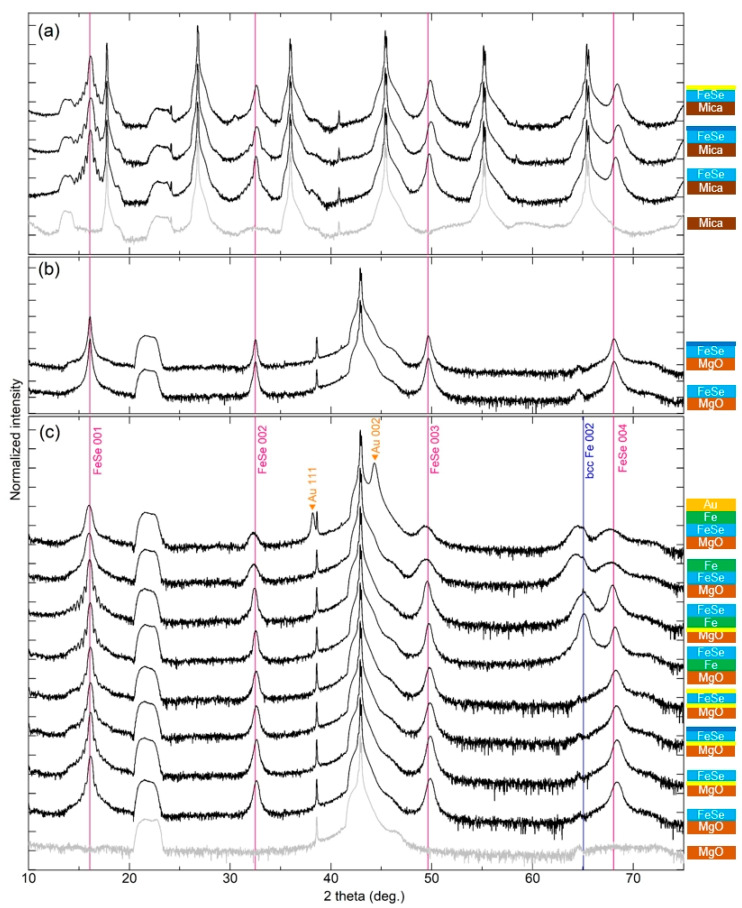
2θ/ω scans of (**a**) FeSe/Mica, (**b**) FeSe/MgO deposited at low growth rates, and (**c**) FeSe/MgO, FeSe/Fe/MgO, Fe/FeSe/MgO, and Au/Fe/FeSe/MgO films with mica and MgO references (shown as grey lines). Intensities were normalized with respect to the MgO (002) reflection in the films deposited on MgO and to mica(003) reflection in the film deposited on mica, respectively. Schematic images of the films corresponding to the neighboring 2θ/ω scans are illustrated at the right side. Yellow lines at the FeSe/MgO and Fe/MgO interfaces represent the MgO substrate pre-annealed in Se vapor. Dark blue and yellow lines at the film surface depict the films post-annealed in UHV and Se vapor, respectively.

**Figure 2:**
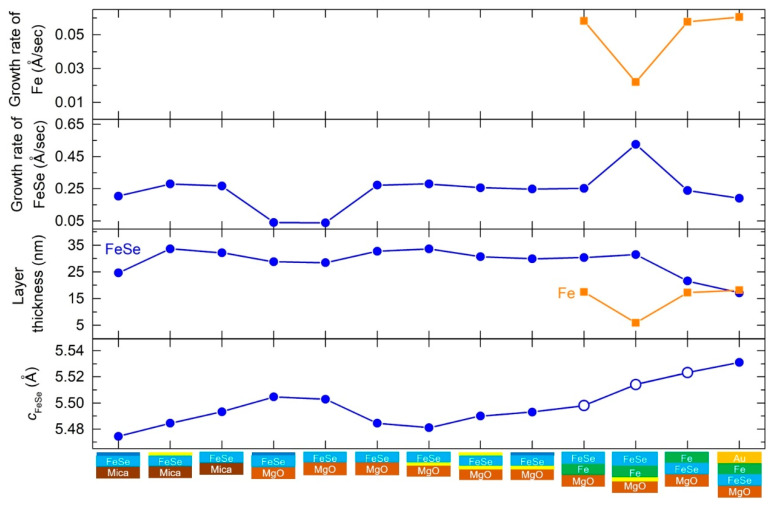
Comparison of *c*-axis of FeSe, *c*_FeSe_, layer thickness, and growth rate of FeSe and Fe among FeSe/mica, FeSe/MgO, FeSe/Fe/MgO, Fe/FeSe/MgO, and Au/Fe/FeSe/MgO films. The schematic images illustrated at the vertical axis represent the films of various preparation conditions. Yellow lines at the FeSe/MgO, Fe/MgO, and FeSe/mica interfaces show the MgO and mica substrates pre-annealed in Se vapor. Dark blue and yellow lines at the film surface depict the films post-annealed in UHV and Se vapor, respectively. Open blue circles in the *c*_FeSe_ window represent the FeSe thin films that show resistive signatures of superconductivity. Please note that most of the films were grown at a similar growth rate of 0.25 Ås^−1^ except the three examples as indicated. Note that the error in the *c*-axis lattice parameter evaluated from reproducibility tests would be ±0.004 Å for FeSe/Fe/MgO and ±0.007 Å for FeSe/MgO.

**Figure 3:**
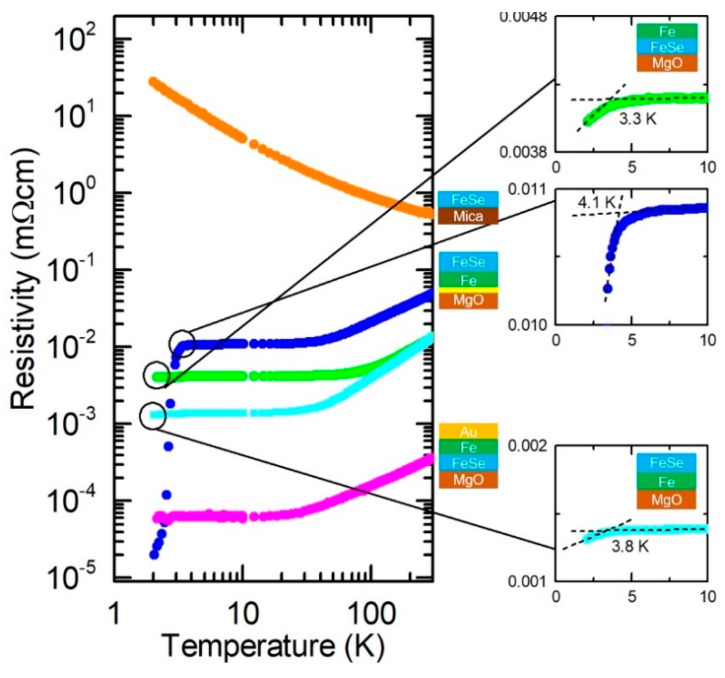
Resistivity vs. temperature (*R* − *T*) curves of an FeSe/Mica film (orange dots) (*T_S_*_,FeSe_ = 350 °C; rep. rate = 10 Hz; *t*_FeSe_ = 32 nm), an FeSe/Fe/MgO film (blue dots) (MgO annealed in Se vapor; *T_S_*,_FeSe_ = 350 °C; *T_S_*_,Fe_ = 650 °C; rep. rate = 10 Hz; *t*_FeSe_ = 32 nm; *t*_Fe_ = 6 nm), an Fe/FeSe/MgO film (light green dots) (MgO annealed in UHV; *T_S_*,_FeSe_ = 350 °C; *T_S_*_,Fe_ at room temperature; rep. rate = 10 Hz; *t*_FeSe_ = 22 nm; *t*_Fe_ = 17 nm), an FeSe/Fe/MgO film (light blue dots) (MgO annealed in UHV; *T_S_*_,FeSe_ = 350 °C; *T_S_*_,Fe_ = 650 °C; rep. rate = 10 Hz; *t*_FeSe_ = 30 nm; *t*_Fe_ = 17 nm), and an Au/Fe/FeSe/MgO (pink dots) (MgO annealed in UHV; *T_S_*_,FeSe_ = 350 °C; *T_S_*_,Fe_ and *T_S_*,_Au_ at room temperature; rep. rate = 10 Hz; *t*_FeSe_ = 17 nm; *t*_Fe_ = 18 nm; *t*_Au_ = 19 nm) at 0 T in logarithmic scale. Expanded insets show the *R* – *T* curves of the Fe/FeSe/MgO and FeSe/Fe/MgO films at low temperatures between 0 and 10 K, where the intersections of dashed lines represent the incipient superconducting transition temperatures, *T_c,onset_*.

Figure 1 summarizes the XRD 2/ω scans for various FeSe films with varying preparation conditions: (1) direct growth on the substrate; (2) growth on the substrate annealed under Se vapor; (3) post-annealed films; (4) FeSe grown on Fe; and (5) capped FeSe films. Schematic images of the films with their various conditions are illustrated next to the figure. Yellow lines at the FeSe/MgO, Fe/MgO, and FeSe/mica interfaces represent the MgO and mica substrates pre-annealed in Se vapor. Dark blue and yellow lines at the film surface depict the films post-annealed in UHV and under Se vapor, respectively. Regardless of the pre- and post-annealing conditions, *c*-axis-oriented, tetragonal FeSe (*t*-FeSe) films were obtained in every film. Figure 2 summarizes *c*- axis of FeSe, *c*_FeSe_, layer thickness and growth rate of FeSe and Fe, and superconducting transition temperature, *T_c_*. In order to assess the variation in the *c*-axis lattice parameter between the conditions in this study, we evaluate the error in the *c*-axis lattice parameter obtained by film reproduction (3 films in a row) to be ±0.004 Å for FeSe/Fe/MgO and ±0.007 Å for FeSe/MgO. Figure 3 summarizes resistivity vs. temperature curves.

### 3.1. FeSe/Mica

Mica has a monoclinic structure without lattice matching to FeSe. Therefore, compared with MgO, it is expected that FeSe grows more relaxed on mica substrates and with less tensile strain. On mica, *c*-axis-oriented FeSe films were obtained with clear Laue fringes around the FeSe (001) reflection, representing a high degree of crystallinity of the FeSe phase (Figure 1a). With a growth rate for FeSe (0.27 Ås^−1^), the *c*-axis lattice parameter, *c*_FeSe_ = 5.493(1) Å, is slightly larger than for films on MgO (Figure 2), consistent with the above-mentioned expectations. The resistivity vs. temperature curve of FeSe/Mica (Figure 3) shows semiconductor-like behavior without any superconductivity transition down to 2 K.

Mica substrates can provide a flexible template to FeSe that may be interesting for generating sensor applications. Previous reports on Fe-Se and FeSe_0.1_Te_0.9_ films on mica substrates show that film bending is possible without cracking of the substrate [26,28], indicating string ductility of the Fe-chalcogenide film. 

### 3.2. Effects of Pre-Annealing and Post-Annealing in UHV and Se Vapor

#### 3.2.1. FeSe/MgO

An important engineering issue in the growth of FeSe thin films is the control of chemical homogeneity at the interface as discussed in Ref. [21]. The heterogeneity of the FeSe/MgO interface with an overall Se deficiency has motivated growth experiments with pretreated substrates under Se vapor. The initial Se deficiency at the film/substrate interface may arise due to the high volatility of Se during the PLD process. To test other possibilities for controlling the interface chemistry apart from growing buffer layers, a substrate pretreatment by Se vapor was performed in the present studies. 

As reported previously [21], 20–30 nm thin FeSe films deposited at a growth rate of ~ 0.25 Ås^−1^ on MgO do not show a superconducting transition. Here, *c*_FeSe_ of FeSe on MgO (MgO annealed in UHV; *T_S_*,_FeSe_ = 350 °C; rep. rate = 10 Hz; *t*_FeSe_ = 33 nm) is 5.484(2) Å and shows a slightly decreased value of 5.480(1) Å when MgO was annealed in Se vapor. A slightly increased *c*_FeSe_ = 5.490(1) Å or 5.493(2) Å are found for films post-annealed in either UHV or Se vapor (Figure 2). 

For comparison, FeSe/MgO films were grown at low growth rates of ~ 0.05 Ås^−1^ (similar to the one reported in Ref. [20]) with and without post-annealing in UHV. XRD shows clear difference between the films grown at different growth rates (Figure 1b). The *c*-axis lattice parameter increases, and the Laue fringes around FeSe(001) disappear. The *c*-axis lattice parameter of *c*_FeSe_ = 5.503(1) Å was obtained for the film without post-annealing and increased to 5.505(1) after post-annealing (Figure 2). 

In terms of electrical conductivity, all grown FeSe/MgO films were insulating. A previous study on FeSe/MgO thin film growth reported that post-annealing at 450 °C for 30 min was effective in inducing a complete superconducting transition in FeSe/MgO films thinner than 6 nm [20]. Interestingly, in the present study, such post annealing effect was not reproduced in the films independent of growth rates. Possible reasons for the negative result could be the island growth leading to a discontinuous microstructure or extremely fast degradation as pointed out in Ref. [20]. There may be also possible modifications with post-annealing conditions for future investigations: extension of annealing time or higher Se vapor pressure.

To investigate the effect of substrate pretreatment on the chemical homogeneity at the interface, we performed AES depth profile analysis. Charging effects during AES experiments were decreased by Au capping of the FeSe/MgO film (MgO annealed in Se vapor; *T_S,_*_FeSe_ = 350 °C; rep. rate = 10 Hz; *t*_FeSe_ = 34 nm; *t*_Au_ = 20 nm). A thicker, continuous FeSe/MgO film (MgO annealed in Se vapor; *T_S__,_*_FeSe_ = 350 °C; rep. rate = 10 Hz; *t*_FeSe_ = 60 nm) was measured as a reference. In both cases, AES depth profiling shows a Se-rich surface, but the film/substrate interface remains Fe-rich, similar to the reported data in Ref. [21].

#### 3.2.2. FeSe/Fe/MgO

FeSe/Fe/MgO films were prepared after substrate annealing either in UHV or in Se vapor. On the Fe buffer, *c*-axis-oriented FeSe films were obtained with *c*_FeSe_ = 5.497(1) and 5.514(2) Å (Figure 2). A larger *c*-axis lattice parameter was obtained in the film grown on the Se-pretreated MgO. In comparison with FeSe/MgO films grown at a growth rate of ~ 0.25 Ås^−1^, more pronounced Laue fringes can be seen around the FeSe (001) reflection (Figure 1c), suggesting a higher degree of crystallinity of the FeSe phase on MgO. In one of the films, the growth rate of FeSe increases by a factor of ~ 2, although the same PLD parameters were used. FeSe/Fe/MgO films show a metallic conductivity and become superconducting. We find an incipient superconducting transition at *T_c_* ≤ 2 K (*T_c,onset_* ≈ 3.3 K) in one film, and a steep superconducting transition at *T_c_* = 3.4 K (*T_c,onset_* ≈ 4.1 K) for the film with substrate pretreated in Se vapor (Figure 3).

Changes in *c*_FeSe_ can be indicative of changes in the anion height, *h*_Se_ from the Fe layer in the FeSe unit cells. *h*_Se_ can be estimated from *h*_Se_ = *c*_FeSe_ × *z*_Se_, where *z*_Se_ denotes the Wyckoff position of Se [29]. Assuming that *z*_Se_ = 0.275 of bulk tetragonal FeSe [30] is the same for that of thin FeSe film, *h*_Se_ would change proportionally to *c*_FeSe_, implying that *h*_Se_ is enhanced by adding the Fe buffer. In a phenomenological point of view, *h*_Se_ has been perceived as a fundamental structural parameter which governs the superconducting transition temperature *T_c_* in Fe-based superconductors [31]. Accordingly, the change in *c*_FeSe_ coincides with those in electrical transport, as depicted in Figure 3, with a superconducting transition in FeSe/Fe/MgO at *T_c_* = 3.4 K. The *T_c_* value is comparable to that reported in Ref. [21]; however, it has a smaller *c*-axis lattice parameter. In future investigations, more detailed structural analysis on *z*_Se_ values is needed to confirm the effects of structural parameters of FeSe/MgO films on superconducting properties.

#### 3.2.3. FeSe/Mica

FeSe films were grown on mica and post-annealed either in UHV (MgO annealed in UHV; *T_S_*,_FeSe_ = 350 °C; rep. rate = 10 Hz; *t*_FeSe_ = 25 nm) or in Se vapor (MgO annealed in UHV; *T_S_*,_FeSe_ = 350 °C; rep. rate = 10 Hz; *t*_FeSe_ = 34 nm). Similar to post-annealed FeSe/MgO films, *c*-axis-oriented FeSe films were obtained with clear Laue fringes at the FeSe(001) reflection (Figure 1a), but with reduced *c*_FeSe_ values: *c*_FeSe_ = 5.474(3) after post-annealing in UHV and 5.484(4) Å after post-annealing in Se vapor, respectively (Figure 2). In contrast to FeSe/MgO where post-annealing led to an increase in *c*_FeSe_, post-annealing of FeSe/mica films resulted in a decrease in *c*_FeSe_ (Figure 2). 

### 3.3. Fe Overlayers: Fe/FeSe/MgO

**Figure 4:**
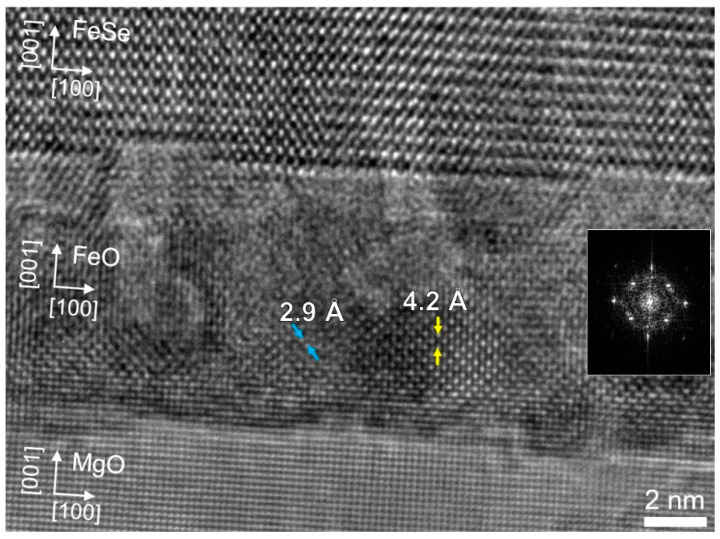
HR TEM image of the FeSe/Fe interface in the FeSe/Fe/MgO film (MgO annealed in UHV; *T_S_*_,FeSe_ = 250 °C; *T_S_*_,Fe_ = 600 °C; rep. rate = 10 Hz; *t*_FeSe_ = 33 nm; *t*_Fe_ = 8 nm). An epitaxial relationship with (001)[100]FeSe//(001)[100]FeO//(001)[100]MgO remained. Inset shows a diffraction pattern of FeO in the particular layer.

Au/Fe/FeSe/MgO and Fe/FeSe/MgO films were annealed in UHV. Similar to the FeSe/MgO and FeSe/Fe/MgO films, *c*-axis-oriented FeSe films were obtained. However, no Laue fringes around the FeSe (001) reflection were found, suggesting less well-ordered lattice planes of the FeSe phase. For the Au-capped film, *c*_FeSe_ increases to 5.531(3) Å, and for Fe/FeSe/MgO *c*_FeSe_ = 5.523(2) Å, as illustrated in Figure 2. Figure 4 also illustrates the resistivity vs. temperature curves of the Au/Fe/FeSe/MgO and Fe/FeSe/MgO films, depicting that both of them were metallic. The Fe/FeSe/MgO film showed an incipient superconducting transition at *T_c_* ≤ 2 K (*T_c,onset_* ≈ 3.8 K), whereas such transition was absent in the Au/Fe/FeSe/MgO film.

It can be seen in Figure 2 that the deposition of Fe on FeSe leads to an increase in *c*_FeSe_, resulting in a comparable value to that in the FeSe/Fe/MgO film. The FeSe/Fe/MgO film shows an incipient superconducting transition above 2 K, possibly denoting the upper limit for the *c*-axis lattice parameter that is favorable for superconductivity. In case of Au/Fe/FeSe/MgO, *c*_FeSe_ = 5.531(3) Å is slightly larger than that of Fe/FeSe/MgO, resulting in a suppressed superconducting transition. These results suggest that structural parameters and electrical transport properties of FeSe can be manipulated in a similar way, independent from the order of FeSe and Fe layers.

In summary, all preparation conditions may have additional statistical variation, which was beyond the scope of the present work. In particular, the effect of the substrate on the structural parameters is much larger than the effect of post-annealing. 

### 3.4. Oxidization of Fe Buffers in FeSe/Fe/MgO

In a previous publication [21], we have demonstrated a chemically and structurally better controlled interface (i.e., between FeSe and Fe) employing an Fe buffer layer replacing the heterogeneous FeSe/MgO interface. Similar to FeSe/MgO films, FeSe/Fe/MgO deteriorate with time when exposed to air. Figure 4 shows a high-resolution (HR) TEM image of the FeSe/Fe interface in FeSe/Fe/MgO (MgO annealed in UHV; *T*_S,FeSe_ = 250 °C; *T*_S,Fe_ = 600 °C; rep. rate = 10 Hz; *t*_FeSe_ = 33 nm; *t*_Fe_ = 8 nm). The film was stored in an evacuated desiccator for ~8 months and in air for ~2 months prior to TEM observations. The TEM analysis revealed partial oxidation of the Fe buffer layer after film storage: FeSe/Fe/MgO converted to FeSe/(Fe+FeO)/MgO, which demonstrates its degradation due to oxidization. This result shows the requirement for appropriate capping layers and protection of the thin film specimens.

## 4. Conclusions

In this study, we address various engineering limitations for the PLD growth of FeSe thin films, which need to be solved for future sensor application: prevention of island growth, control of homogeneous film/substrate interface, and protection from fast surface degradation. We have tested the deposition of ~30 nm thin films on mica, MgO, and Fe/MgO in addition to substrate pretreatment and post-annealing of the films. Furthermore, we have also tested Fe overlayers and Au capping. Many aspects, which have been addressed for the first time here, still require optimized analysis. Resistive signatures of superconductivity were only found in FeSe/Fe/MgO as well as in Fe/FeSe/MgO. Apart from technical issues revealed in the thin film growth of FeSe by pulsed laser deposition, it is promising that *c*-axis-oriented, tetragonal FeSe is confirmed to grow on such substrates as mica, which is a bendable material favorable for flexible device fabrication. Our work provides for sensor application studies on FeSe a preliminary investigation for selection of effective engineering protocols and further optimization.

## Figures and Tables

**Table 1:** Relevant literature for growth of FeSe/MgO films.

*T_c 90_* (K)	Thickness (nm)	Comment	Composition		Method	Ref.
			Target	Film		
N/A	400	With secondary phase Fe_7_Se_8_	Fe_1−x_Cu_x_Se_1−x/2_^4^	FeSe, Fe_7_Se_8_	PLD	6
no SC ^1^	<20	N/A	FeSe ^4^	N/A	RF sputtering	8
no SC ^1^	50	epitaxial	N/A	N/A	PLD	22
<2	>140	Two domains	FeSe ^4^	FeSe_1−x_	PLD	4
<4	18	N/A	FeSe ^4^	FeSe	PLD & post-annealing	20
~5	29	N/A	FeSe ^4^	N/A	RF sputtering	8
~5	400	epitaxial	FeSe	FeSe_1+x_	PLD	5
~7	200	epitaxial	N/A	N/A	PLD	22
<2(LT) ^2^ 7 (HT) ^3^	140	Different epitaxy for LT and HT	FeSe_1−x_	FeSe_1−x_	PLD	23
~8	160	N/A	FeSe_0.95_	N/A	PLD	19

^1^ Superconducting transition ^2^ Deposited at low temperature (LT) ^3^ Deposited at high temperature (HT) ^4^ Nominal values.

## Data Availability

All data are available upon request to all corresponding authors.
